# Metabolic Analysis of Various Date Palm Fruit (*Phoenix dactylifera* L.) Cultivars from Saudi Arabia to Assess Their Nutritional Quality

**DOI:** 10.3390/molecules200813620

**Published:** 2015-07-27

**Authors:** Ismail Hamad, Hamada AbdElgawad, Soad Al Jaouni, Gaurav Zinta, Han Asard, Sherif Hassan, Momtaz Hegab, Nashwa Hagagy, Samy Selim

**Affiliations:** 1 Department of Clinical Laboratory Sciences, College of Applied Medical Sciences, Aljouf University, Sakaka 2014, Saudi Arabia; E-Mails: ismailhamad@yahoo.com (I.H.); abood127@yahoo.com (S.H.); 2 Laboratory for Molecular Plant Physiology and Biotechnology, Department of Biology, University of Antwerp, Groenenborgerlaan 171, B-2020, Antwerp 2020, Belgium; E-Mails: hamada.abdelgawad@uantwerpen.be (H.A.); gaurav.zinta@uantwerpen.be (G.Z.); han.asard@uantwerpen.be (H.A.); 3 Department of Botany, Faculty of Science, University of Beni-Suef, Beni-Suef 62511, Egypt; E-Mail: momtazyehya@hotmail.com; 4 YAJ Prophatic Medicine Application, College of Medicine, King Abdulaziz University, P.O. Box 80215, Jeddah 21589, Saudi Arabia; E-Mail: saljaouni@kau.edu.sa; 5 Microbiology and Botany Department, Faculty of Science, Suez Canal University, Ismailia 41522, Egypt; E-Mail: nashwa_hagag@hotmail.com; 6 Biochemistry Department, Bahri University, Khartoum 1660, Sudan

**Keywords:** date palm, lipid peroxidation, antioxidants, antimicrobial, minerals, phenolics, amino acids, organic acids, metabolomics

## Abstract

Date palm is an important crop, especially in the hot-arid regions of the world. Date palm fruits have high nutritional and therapeutic value and possess significant antibacterial and antifungal properties. In this study, we performed bioactivity analyses and metabolic profiling of date fruits of 12 cultivars from Saudi Arabia to assess their nutritional value. Our results showed that the date extracts from different cultivars have different free radical scavenging and anti-lipid peroxidation activities. Moreover, the cultivars showed significant differences in their chemical composition, e.g., the phenolic content (10.4–22.1 mg/100 g DW), amino acids (37–108 μmol·g^−1^ FW) and minerals (237–969 mg/100 g DW). Principal component analysis (PCA) showed a clear separation of the cultivars into four different groups. The first group consisted of the Sokary, Nabtit Ali cultivars, the second group of Khlas Al Kharj, Khla Al Qassim, Mabroom, Khlas Al Ahsa, the third group of Khals Elshiokh, Nabot Saif, Khodry, and the fourth group consisted of Ajwa Al Madinah, Saffawy, Rashodia, cultivars. Hierarchical cluster analysis (HCA) revealed clustering of date cultivars into two groups. The first cluster consisted of the Sokary, Rashodia and Nabtit Ali cultivars, and the second cluster contained all the other tested cultivars. These results indicate that date fruits have high nutritive value, and different cultivars have different chemical composition.

## 1. Introduction

Date palm (*Phoenix dactylifera* L.) is the most successful and commercially important crop in the hot-arid regions of the world, e.g., Saudi Arabia, Emirates and Egypt [1,2]. In these countries, date palm products are commonly used for human and animal consumption, pharmaceuticals, cosmetics, carpentry, and firewood. A large number of date palm cultivars are known, however, until now only a few of these cultivars have been evaluated for chemical composition and nutritional quality [1,2].

Date fruits have a great importance from both a nutritional and therapeutic point of view [3,4]. They are rich sources of sugars, vitamins, minerals and fibers. In some date varieties, the sugar content of the fruits reaches up to 88%, and such fruits are considered a high-energy food source [5]. Moreover, date fruits possess antioxidant and antimutagenic properties [6,7], attributable to their high levels of polyphenolic compounds and vitamins [7,8]. For example, Al-Farsi *et al.* [9] found that total phenolic content ranged from 172 to 246 mg of gallic acid/100 g in three date varieties grown in Oman. Yousif *et al.* [10] observed that date fruits have high vitamin levels, e.g., ascorbic acid (2.4–17.5 mg/100 g), thiamine (0.08–0.13 mg/100 g) and riboflavin (0.13–17.5 mg/100 g). Moreover, dates are rich in dietary fiber (6.4%–11.5%), which further improves their nutritional value and therapeutic utility [5,11]. Extracts of dates also show antibacterial and antifungal properties [12,13,14]. Considering the nutritional importance of dates, studying their biochemical composition and nutritional quality is increasingly being recognized as a worthy and important task.

Varying growth conditions cause changes in the primary and secondary metabolism of plants [15,16,17,18,19,20,21,22]. Similarly, the growth conditions of different regions induce several external and internal changes in the dates. Such changes are often classified on the basis of observed differences in the color and chemical composition of date fruits. For instance, the nutritional quality of dates varies among varieties grown in Algeria [7], Egypt [23], Oman [24,25] and Bahrain [26]. The study of Farag *et al.* [23] recorded a high variation in the chemical metabolites of 21 Egyptian date varieties. In particular their study revealed that the phenolic contents varied considerably among these varieties [2.3–19 g·kg−1 DW]. Moreover, cluster analyses indicated that flavonols and sugars both contribute the most to variety classification. Recently, metabolomics have been successfully applied to investigate the chemical composition of dates to assess their nutritional quality [27,28,29], and such approaches are crucial to establish links between plant genotypes and phenotypes.

The aim of this study was to evaluate the biological activity and nutritional quality of 12 date varieties from different geographical locations in Saudi Arabia. These varieties correspond to the commonly used ones in this region. In this study, we used high performance liquid chromatography (HPLC) coupled to electrochemical and diode array detection and mass spectrometry (HPLC/PDA/MS). These techniques enabled us to analyze a wide range of metabolites including sugars, amino acids, fatty acids, organic acids, phenolics and flavonoids, and antioxidants in the 12 different palm varieties. We also assessed the macro- and microelemental mineral profile and hydrophilic and lipophilic antioxidant contents of these cultivars. To acquire statistical correlations among all the measured parameters and different palm cultivars, we performed principle component analysis (PCA) and hierarchical clustering analysis (HCA).

## 2. Results and Discussion

### 2.1. Metabolites

#### 2.1.1. Antioxidants

**Figure 1:**
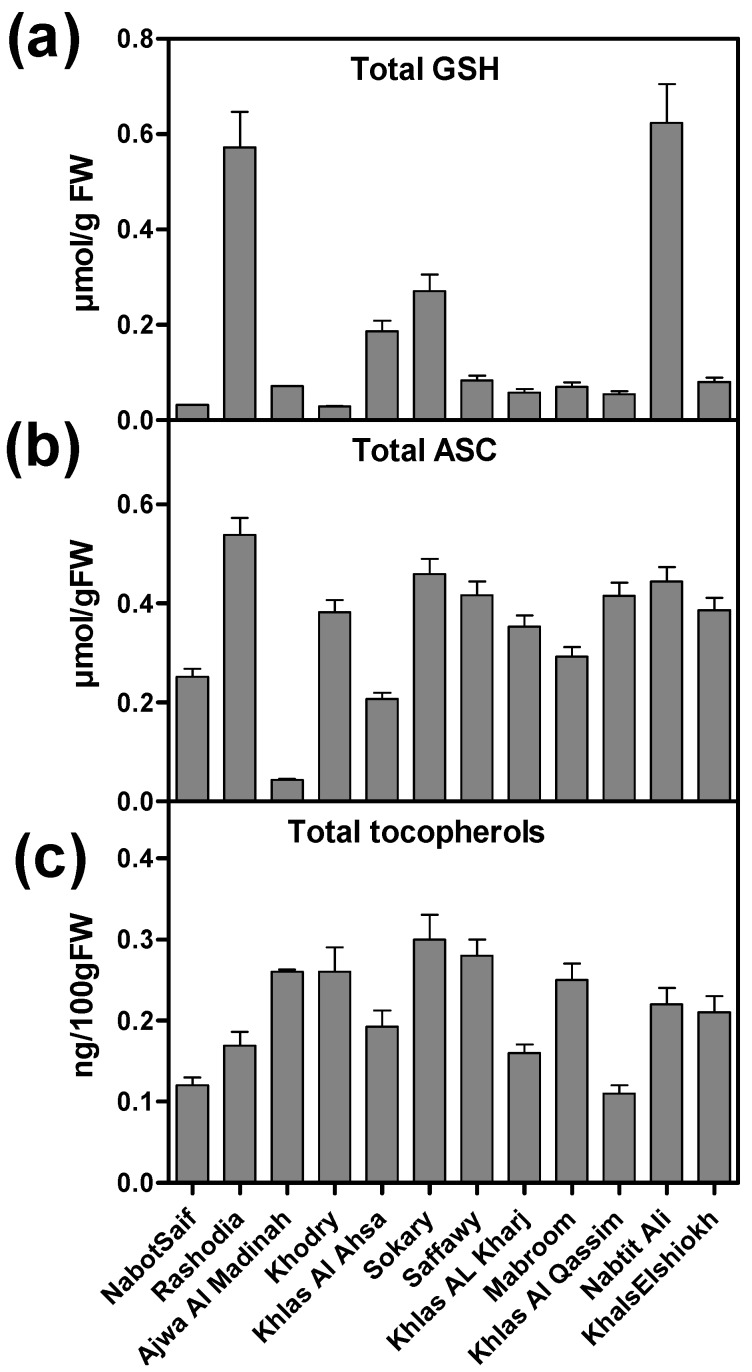
Antioxidant metabolites content: (**a**) Total glutathione (GSH); (**b**) total ascorbate (ASC); (**c**) total tocopherols of 12 Saudi date cultivars.

Glutathione (GSH) and ascorbic acid (ASC) are aqueous phase antioxidants, while tocopherol is lipophilic in nature. GSH content showed a variation among the cultivars, ranging from 0.011 to 0.295 μmol·g^−1^ FW (Figure 1, Table 1). The highest GSH content was observed for the Rashodia, Khlas Al Ahsa and Nabtit Ali cultivars (0.247, 0.177 and 0.295 μmol·g^−1^ FW, respectively) while Khodry had the lowest GSH content (0.011 μmol·g^−1^ FW). Similarly, ASC content varied significantly among the date cultivars and was in the range of 0.051 and 0.541 μmol·g^−1^ FW (Table 1). Rashodia, Sokary and Nabtit Ali showed the highest ASC content (0.541, 0.526 and 0.516 μmol·g^−1^ FW respectively) and Ajwa Al Madinah showed the lowest ASC content (0.051 μmol·g^−1^ FW) (Table 1). Total tocopherol content was in the range of 0.09 to 0.28 μmol·g^−1^ FW (Table 1) where Sokary had the highest content (0.28 μmol·g^−1^ FW), and Khla Al Qassim had the lowest tocopherol content (0.09 μmol·g^−1^ FW) (Table 1). The redox ratios of GSH and ASC, and α-, β-, γ- and δ-tocopherols are listed in Table 1.

#### 2.1.2. Amino Acids

Many amino acids were detected in the fruits of the twelve studied cultivars, which were rich in amino acids. Moreover, the cultivars showed significant differences in their amino acids contents (37–108 μmol·g^−1^ FW). Proline was the major amino acid, and it was highly abundant in the Nabitit Ali and Rashodia cultivars (85 and 126 μmol·g^−1^ FW, respectively). On the other hand, cysteine was the minor amino acid (0.001–0.11 μmol·g^−1^ FW) (Table 2).

**Table 1:** Concentrations of glutathione (GSH), ascorbate (ASC) and tocopherols (toco), and redox status of GSH and ASC in 12 Saudi date cultivars.

Cultivars	GSH (μmol·g^−1^ FW)	GSH Redox Status (%)	ASC (μmol·g^−1^ FW)	ASC Redox Status (%)	Alfa Toc. (ng/100 g FW)	Beta Toc. (ng/100 g FW)	Gamma Toc. (ng/100 g FW)	Delta Toc. (ng/100 g FW)
Nabot Saif	0.025 ± 0.003	77.366 ± 5.532	0.254 ± 0.023	99.259 ± 2.637	0.086 ± 0.009	0.023 ± 0.002	0.016 ± 0.001	0 ± 0.0
Rashodia	0.247 ± 0.026	43.523 ± 5.111	0.541 ± 0.049	99.643 ± 2.646	0.124 ± 0.013	0.013 ± 0.002	0.021 ± 0.002	0 ± 0.0
Ajwa Al Madinah	0.062 ± 0.007	86.049 ± 5.794	0.051 ± 0.005	85.244 ± 3.061	0.212 ± 0.022	0.022 ± 0.002	0.022 ± 0.003	0.003 ± 0.00
Khodry	0.011 ± 0.001	39.373 ± 4.728	0.387 ± 0.035	98.891 ± 3.551	0.197 ± 0.02	0.033 ± 0.002	0.044 ± 0.004	0.002 ± 0.00
Khlas Al Ahsa	0.177 ± 0.018	96.507 ± 22.175	0.206 ± 0.019	100.791 ± 3.619	0.14 ± 0.014	0.026 ± 0.002	0.016 ± 0.001	0.010 ± 0.001
Sokary	0.059 ± 0.006	21.736 ± 0.580	0.526 ± 0.047	87.570 ± 2.326	0.218 ± 0.022	0.019 ± 0.0021	0.043 ± 0.005	0.011 ± 0.001
Saffawy	0.039 ± 0.005	48.128 ± 5.652	0.423 ± 0.038	98.662 ± 2.621	0.179 ± 0.0188	0.023 ± 0.0024	0.038 ± 0.0	0.04 ± 0.002
Khlas Al Kharj	0.054 ± 0.006	94.605 ± 11.110	0.346 ± 0.031	100.261 ± 2.717	0.113 ± 0.0177	0.027 ± 0.0024	0.018 ± 0.001	0.003 ± 0.0
Mabroom	0027 ± 0.003	38.279 ± 1.021	0.296 ± 0.027	99.577 ± 14.785	0.197 ± 0.02	0.014 ± 0.0018	0.023 ± 0.002	0.008 ± 0.00
Khla Al Qassim	0.039 ± 0.004	72.546 ± 1.935	0.421 ± 0.038	99.408 ± 14.760	0.072 ± 0.007	0.025 ± 0.001	0.0157 ± 0.003	0.001 ± 0.00
Nabtit AIi	0.295 ± 0.031	47.527 ± 1.267	0.516 ± 0.049	86.407 ± 6.863	0.158 ± 0.0165	0.020 ± 0.0012	0.038 ± 0.004	0 ± 0.00
Khals El Shiokh	0.065 ± 0.007	82.268 ± 2.194	0.376 ± 0.034	100.600 ± 15.382	0.163 ± 0.017	0.019 ± 0.0021	0.016 ± 0.002	0.005 ± 0.00
*p* value	0	0	0	0.093	0	0.154	0	0

**Table 2:** Concentrations of amino acids (μmol·g^−1^ FW) in 12 Saudi date cultivars.

Cultivars	Proline	Glycine	Lysine	Histidine	Alanine	Arginie	Ornithine	Glutamine	Asparagine	Isoleucine
Nabot Saif	76 ± 11	78 ± 8.8	3.8 ± 0.6	1.16 ± 0.18	19.2 ± 2.1	0.43 ± 0.0	0.03 ± 0.00	0.41 ± 0.04	0.72 ± 0.07	0.10 ± 0.01
Rashodia	85 ± 13	39 ± 4.5	2.9 ± 0.4	0.84 ± 0.13	11.3 ± 1.2	2.7 ± 0.3	0.13 ± 0.01	1.61 ± 0.16	1.101 ± 0.1	0.15 ± 0.01
Ajwa Al Madinah	16 ± 2.6	65 ± 7.4	7.3 ± 1.1	0.99 ± 0.1	9.2 ± 1.0	1.42 ± 0.1	0.15 ± 0.01	1.02 ± 0.1	0.26 ± 0.03	0.15 ± 0.01
Khodry	11 ± 1.7	57 ± 6.5	3.2 ± 0.5	0.98 ± 0.1	8.07 ± 0.9	0.31 ± 0.0	0.042 ± 0.0	0.14 ± 0.01	1.07 ± 0.1	0.09 ± 0.0
Khlas Al Ahsa	14 ± 2.2	75 ± 8.5	4.4 ± 0.6	1.47 ± 0.2	12.8 ± 1.4	0.24 ± 0.0	0.13 ± 0.02	0.56 ± 0.05	0.518 ± 0.2	0.15 ± 0.05
Sokary	12 ± 19	13 ± 1.5	2.2 ± 0.3	1.40 ± 0.2	5.8 ± 0.64	1.11 ± 0.1	0.1 ± 0.01	1.20 ± 0.1	4.4 ± 0.4	1.79 ± 0.17
Saffawy	28 ± 4.3	49 ± 5.5	3.2 ± 0.5	0.97 ± 0.1	11.5 ± 1.2	0.30 ± 0.0	0.038 ± 0.0	0.30 ± 0.03	1.2 ± 0.1	0.08 ± 0.00
Khlas Al Kharj	8 ± 1.3	49 ± 5.6	3.4 ± 0.5	1.20 ± 0.2	7.6 ± 0.8	0.43 ± 0.0	0.1 ± 0.02	0.30 ± 0.03	0.07 ± 0.0	0.13 ± 0.01
Mabroom	10 ± 1.5	57 ± 6.5	4.52 ± 0.	0.07 ± 0.01	13.6 ± 1.5	0.21 ± 0.0	0.021 ± 0.0	0.43 ± 0.01	0.93 ± 0.08	0.081 ± 0.0
Khla Al Qassim	9.5 ± 1.5	47 ± 5.3	1.9 ± 0.3	0.91 ± 0.14	16 ± 1.8	0.99 ± 0.1	0.06 ± 0.01	0.25 ± 0.02	0.09 ± 0.0	0.11 ± 0.01
Nabtit AIi	126 ± 6	17 ± 1.9	1.0 ± 0.0	0.97 ± 0.15	7.07 ± 0.7	2.65 ± 0.2	0.13 ± 0.0	3.5 ± 0.34	1.4 ± 0.15	1.39 ± 0.14
Khals El Shiokh	10.3 ± 1.6	38 ± 4.3	3.0 ± 0.4	1.09 ± 0.17	13.2 ± 1.5	0.50 ± 0.0	0.09 ± 0.01	0.48 ± 005	0.97 ± 0.1	0.15 ± 0.01
*p* value	0.0	0.0	0.0	0.0	0.0	0.0	0.0	0.0	0.0	0.0
**Cultivars**	**Leucine**	**Methionine**	**Threonine**	**Valine**	**Serine**	**Phenylalanine**	**Glutamic acid**	**Cysteine**	**Tyrosine**	
Nabot Saif	0.014 ± 0.0	0.012 ± 0.00	0.074 ± 0.0	1.157 ± 0.2	0.13 ± 0.0	0.38 ± 0.05	1.0 ± 0.18	0.01 ± 0.0	0.462 ± 0.05	
Rashodia	0.018 ± 0.0	0.016 ± 0.0	0.112 ± 0.01	0.93 ± 0.17	0.19 ± 0.0	0.19 ± 0.03	0.7 ± 0.13	0.02 ± 0.0	0.39 ± 0.04	
Ajwa Al Madinah	0.02 ± 0.00	0.021 ± 0.00	0.027 ± 0.0	3.13 ± 0.6	0.19 ± 0.0	0.99 ± 0.14	0.8 ± 0.15	0.001 ± 0.0	0.80 ± 0.08	
Khodry	0.1 ± 0.01	0.09 ± 0.01	0.110 ± 0.01	1.188 ± 0.2	0.11 ± 0.0	0.36 ± 0.05	0.8 ± 0.15	0.009 ± 0.0	0.35 ± 0.00	
Khlas Al Ahsa	0.06 ± 0.00	0.05 ± 0.00	0.053 ± 0.00	0.80 ± 0.15	0.19 ± 0.0	0.70 ± 0.1	1.3 ± 0.22	0.001 ± 0.0	0.94 ± 0.1	
Sokary	0.19 ± 0.02	0.173 ± 0.02	0.45 ± 0.05	0.493 ± 0.09	2.20 ± 0.2	0.43 ± 0.07	1.2 ± 0.2	0.16 ± 0.01	0.74 ± 0.08	
Saffawy	0.07 ± 0.00	0.067 ± 0.0	0.12 ± 0.01	0.71 ± 0.13	0.11 ± 0.0	0.11 ± 0.02	0.8 ± 0.1	0.007 ± 0.0	0.06 ± 0.0	
Khlas Al Kharj	0.25 ± 0.02	0.22 ± 0.02	0.0074 ± 0.0	0.87 ± 0.17	0.17 ± 0.0	0.32 ± 0.05	1.0 ± 0.1	0.007 ± 0.0	0.63+0.07	
Mabroom	0.07 ± 0.00	0.064 ± 0.00	0.095 ± 0.01	0.81 ± 0.15	0.10 ± 0.0	0.44 ± 0.07	0.06 ± 0.0	0.007 ± 0.0	0.42 ± 0.05	
Khla Al Qassim	0.082 ± 0.0	0.072 ± 0.00	0.009 ± 0.00	0.47 ± 0.08	0.13 ± 0.0	0.18 ± 0.03	0.79 ± 0.1	0.15 ± 0.02	0.43 ± 0.05	
Nabtit AIi	0.084 ± 0.0	0.074 ± 0.00	0.15 ± 0.01	0.69 ± 0.13	1.72 ± 0.1	0.12 ± 0.02	0.85 ± 0.1	0.11 ± 0.01	0.48 ± 0.05	
Khals El Shiokh	0.082 ± 0.0	0.071 ± 0.01	0.09 ± 0.01	0.65 ± 0.1	0.18 ± 0.0	0.27 ± 0.04	0.95 ± 0.17	0.11 ± 0.01	0.613 ± 0.07	
*p* value	0.0	0.0	0.0	0.0	0.0	0.0	0.0	0.0	0.0	

#### 2.1.3. Sugars

We measured monosaccharides (glucose, fructose), disaccharides (sucrose) and total sugar content in the 12 date cultivars (Table 3). The total sugar content in the date cultivars was quite high, e.g., Khla Al Qassim had 0.11 mg/100 g FW and 0.3 mg/100 g FW, suggesting that date fruit are rich sources of sugars. Most of the studied cultivars had higher glucose and fructose concentrations, conversely Nabtit Ali, Sokary and Rashodia cultivars has higher sucrose levels (Table 3).

#### 2.1.4. Organic Acids

The average content of total organic acids in dates of tested varieties were 17 to 26 mg·g^−1^ FW. Six organic acids were identified, among which malic acid was the predominant organic acid, and its concentration ranged from 5 to 10 mg·g^−1^ DW, followed by lesser amounts of succinic acid, isobutyric acid, citric acid, oxalic acid and formic acid (Table 3).

**Table 3:** Concentrations of sugars (mg/100 g FW) and organic acids (mg·g^−1^ FW) in 12 Saudi date cultivars.

Cultivars	Glucose	Fructose	Sucrose	Oxalic	Malic	Succinic	Citric	Isobutyric	Formic
Nabot Saif	50.1 ± 0.0	58.8 ± 1.8	26.55 ± 0.0	1.93 ± 0.11	9.66 ± 0.58	1.59 ± 0.08	2.70 ± 0.14	2.34 ± 0.12	0.29 ± 0.02
Rashodia	42.5 ± 0.6	53.0 ± 0.0	112.5 ± 0.0	1.64 ± 0.1	7.03 ± 0.79	5.69 ± 0.7	2.86 ± 0.38	2.88 ± 0.15	0.37 ± 0.02
Ajwa Al Madinah	35.4 ± 0.5	39.4 ± 2.5	13.45 ± 0.2	1.46 ± 0.09	10.12 ± 1.18	0.76 ± 0.08	2.01 ± 0.23	3.12 ± 0.19	0.35 ± 0.02
Khodry	58.1 ± 0.0	69.16 ± 2.1	19.42 ± 0.0	2.24 ± 0.13	11.41 ± 0.69	1.19 ± 0.07	2.31 ± 0.22	2.09 ± 0.11	0.32 ± 0.02
Khlas Al Ahsa	58.2 ± 3.6	74.1 ± 4.7	17.9 ± 0.27	2.73 ± 0.17	13.98 ± 0.85	1.23 ± 0.08	2.33 ± 0.14	2.01 ± 0.12	0.17 ± 0.01
Sokary	1.5 ± 1.8	59.5 ± 3.7	138.5 ± 5.0	2.18 ± 0.13	10.43 ± 0.55	9.26 ± 0.56	4.65 ± 0.25	2.94 ± 0.18	0.29 ± 0.02
Saffawy	47.3 ± 0.07	54.26 ± 2.4	28.7 ± 1.04	1.82 ± 0.1	9.10 ± 0.47	1.86 ± 0.11	0.95 ± 0.05	3.23 ± 0.18	0.21 ± 0.01
Khlas Al Kharj	95.40 ± 0.0	112.7 ± 3.4	31.9 ± 0.0	1.90 ± 0.1	17.68 ± 1.08	1.82 ± 0.09	0.93 ± 0.05	3.30 ± 0.17	0.27 ± 0.02
Mabroom	46.30 ± 0.70	62.0 ± .00	20.1 ± 0.0	1.85 ± 0.1	8.68 ± 0.88	1.07 ± 0.13	2.14 ± 0.26	2.62 ± 0.31	0.17 ± 0.02
Khla Al Qassim	79.6 ± 0.0	101.2 ± 0.0	26.1 ± 0.0	1.57 ± 0.08	13.20 ± 1.76	1.40 ± 0.16	2.41 ± 0.27	2.07 ± 0.23	0.20 ± 0.03
Nabtit AIi	21.08 ± 0.3	23.20 ± 1.47	150.5 ± 2.2	0.83 ± 0.04	10.01 ± 1.1	8.66 ± 0.82	4.43 ± 0.42	2.16 ± 0.22	0.23 ± 0.03
Khals El Shiokh	58.2 ± 0.0	71.29 ± 2.2	9.23 ± 0.0	2.49 ± 0.15	12.94 ± 0.78	0.62 ± 0.04	1.98 ± 0.12	1.70 ± 0.1	0.19 ± 0.01
*p* value	0.01	0	0.05	0	0	0	0	0	0

#### 2.1.5. Phenolics and Flavonoids

We recorded high total phenolic contents, in the range of 10.47 to 22.11 mg/100 g FW. In details, Ajwa Al Madinah had the highest content (22.11 mg/100 g DW), followed by Nabt Saif (22 mg/100 g DW), while Khla Al Qassim had the lowest content (10.47 mg/100 g DW). Differences (*p* < 0.05) in total content of phenolics were observed among date varieties (Table 4). In this study, gallic, *p*-coumaric, and ferulic acid derivatives were the most dominant phenolic compounds, respectively. Moreover, different classes of flavonoids were identified in the tested varieties; quercetin, luteolin, apigenin, isoquercetrin, and rutin. Total flavonoid content was in the range of 1.22 and 2.82 mg/100 g DW, whereas Saffawy had the highest content (2.82 mg/100 g DW), followed by Ajwa Al Madinah (2.78 mg/100 g DW), and Al Qassim had the lowest content 1.22 mg/100 g DW (Table 5).

#### 2.1.6. Elemental Profiling (Macro- and Micronutrients)

Our tested date cultivars contained significant amounts of minerals (Table 6). In particular, the potassium content was the highest (180.7–796.7 mg/100 g DW), followed in descending order by phosphorus (30.4–110.1 mg/100 g), magnesium (21.1–97.3 mg/100 g), and sodium (4.39–9.37 mg/100 g). Most of the analyzed minerals showed significant differences among the different cultivars; Khlas Al Kharj had the highest content of potassium (796.7 mg/100 g), magnesium (97.3 mg/100 g), sodium (9.3 mg/100 g) and calcium (0.919 mg/100 g) as shown in Table 6, while Nabtit Ali and Sokary have the highest iron contents 1.648 and 1.644 mg/100 g, respectively.

**Table 4:** Concentrations of phenolic compounds (mg/100 g DW) in 12 Saudi date cultivars.

Cultivars	Caffeic acid	Ferulic acid	Protocatechuic acid	Catechin	Gallic acid	*p*-Coumaric acid	Resorcinol	Chlorogenic acid	Syringic acid	Total phenolic
Nabot Saif	0.018 ± 0.004	1.94 ± 0.42	0.162 ± 0.028	0.574 ± 0.12	15.227 ± 3.3	3.275 ± 0.720	0.033 ± 0.007	0.2 ± 0.044	0.58 ± 0.6	22.00 ± 5.35
Rashodia	0.013 ± 0.001	1.44 ± 0.09	0.115 ± 0.007	0.426 ± 0.02	11.312 ± 0.7	2.433 ± 0.154	0.025 ± 0.002	0.149 ± 0.009	0.66 ± 0.0	16.58 ± 1.05
Ajwa Al Madinah	0.026 ± 0.001	2.52 ± 0.11	1.217 ± 0.057	0.526 ± 0.02	13.973 ± 0.6	3.087 ± 0.004	0.030 ± 0.002	0.184 ± 0.009	0.82 ± 0.0	22.11 ± 1.10
Khodry	0.024 ± 0.005	2.56 ± 0.57	1.094 ± 0.243	0.473 ± 0.10	12.564 ± 2.7	2.702 ± 0.601	0.028 ± 0.006	0.165 ± 0.037	0.63 ± 0.2	20.13 ± 4.21
Khlas Al Ahsa	0.018 ± 0.004	1.94 ± 0.42	0.527 ± 0.588	0.353 ± 0.07	9.370 ± 2.05	2.015 ± 0.443	0.021 ± 0.005	0.123 ± 0.027	0.55 ± 0.1	14.92 ± 3.75
Sokary	0.019 ± 0.003	2.01 ± 0.34	0.893 ± 0.119	0.386 ± 0.05	10.24 ± 1.36	2.309 ± 0.324	0.022 ± 0.004	0.135 ± 0.018	0.60 ± 0.0	17.10 ± 2.84
Saffawy	0.026 ± 0.001	2.52 ± 0.11	1.217 ± 0.057	0.526 ± 0.02	13.973 ± 0.6	3.005 ± 0.142	0.030 ± 0.002	0.184 ± 0.009	0.82 ± 0.0	21.99 ± 1.27
Khlas Al Kharj	0.024 ± 0.005	2.56 ± 0.57	1.094 ± 0.243	0.333 ± 0.09	8.829 ± 2.48	1.302 ± 0.290	0.013 ± 0.003	0.080 ± 0.018	0.74 ± 0.1	14.97 ± 1.28
Mabroom	0.018 ± 0.004	1.94 ± 0.42	0.527 ± 0.588	0.353 ± 0.07	9.370 ± 2.05	0.971 ± 0.213	0.010 ± 0.002	0.059 ± 0.013	0.55 ± 0.1	13.80 ± 3.50
Khla Al Qassim	0.013 ± 0.001	1.44 ± 0.09	0.606 ± 0.038	0.262 ± 0.01	6.9610.441	0.721 ± 0.046	0.008 ± 0.001	0.044 ± 0.003	0.41 ± 0.0	10.47 ± 0.63
Nabtit Ali	0.019 ± 0.003	2.01 ± 0.34	0.893 ± 0.119	0.386 ± 0.05	10.246 ± 1.3	1.062 ± 0.141	0.011 ± 0.001	0.065 ± 0.009	0.60 ± 0.0	15.80 ± 2.69
Khals El Shiokh	0.026 ± 0.001	2.52 ± 0.11	1.217 ± 0.057	0.526 ± 0.02	13.973 ± 0.6	1.448 ± 0.068	0.015 ± 0.001	0.089 ± 0.004	0.82 ± 0.0	20.37 ± 1.17
*p* value	0.00	0.00	0.00	0.00	0.00	0.00	0.00	0.00	0.049	0.00

**Table 5:** Concentrations of flavonoid compounds (mg/100 g DW) in 12 Saudi date cultivars.

Cultivars	Quercetin	Luteolin	Apigenin	Isoquercetrin	Rutin	Total Flavonoid
Nabot Saif	0.170 ± 0.020	0.045 ± 0.010	0.291 ± 0.064	0.726 ± 0.160	0.943 ± 0.207	2.175 ± 0.461
Rashodia	1.001 ± 0.063	0.033 ± 0.002	0.216 ± 0.014	0.540 ± 0.034	0.701 ± 0.044	2.491 ± 0.158
Ajwa Al Madinah	1.219 ± 0.071	0.041 ± 0.002	0.263 ± 0.015	0.411 ± 0.001	0.853 ± 0.049	2.787 ± 0.138
Khodry	1.112 ± 0.247	0.026 ± 0.007	0.240 ± 0.053	0.360 ± 0.080	0.547 ± 0.154	2.284 ± 0.219
Khlas Al Ahsa	0.536 ± 0.597	0.028 ± 0.006	0.179 ± 0.039	0.268 ± 0.059	0.580 ± 0.128	1.591 ± 0.366
Sokary	0.838 ± 0.025	0.028 ± 0.001	0.181 ± 0.005	0.271 ± 0.008	0.665 ± 0.093	1.983 ± 0.104
Saffawy	1.270 ± 0.002	0.041 ± 0.002	0.263 ± 0.015	0.394 ± 0.023	0.853 ± 0.049	2.821 ± 0.088
Khlas Al Kharj	1.112 ± 0.247	0.026 ± 0.007	0.081 ± 0.023	0.173 ± 0.039	0.547 ± 0.154	1.939 ± 0.102
Mabroom	0.536 ± 0.597	0.028 ± 0.006	0.086 ± 0.019	0.129 ± 0.028	0.580 ± 0.128	1.359 ± 0.778
Khla Al Qassim	0.616 ± 0.039	0.020 ± 0.001	0.064 ± 0.004	0.096 ± 0.006	0.431 ± 0.027	1.228 ± 0.078
NabtitAIi	0.950 ± 0.133	0.028 ± 0.001	0.087 ± 0.003	0.346 ± 0.049	0.665 ± 0.093	2.076 ± 0.272
Khals El Shiokh	1.219 ± 0.071	0.041 ± 0.002	0.127 ± 0.007	0.443 ± 0.026	0.853 ± 0.049	2.683 ± 0.155
*p* value	0.00	0.00	0.00	0.00	0.00	0.00

**Table 6:** Concentrations of minerals (mg/100 g DW) in 12 Saudi date cultivars.

Cultivars	K	Ca	Mg	P	Na	Cu	Fe	Mn	Cd	Zn
Nabot Saif	431.88 ± 27	0.480 ± 0.042	50.814 ± 3.09	68.603 ± 4.65	5.48 ± 0.609	0.66 ± 0.053	0.27 ± 0.022	0.245 ± 0.016	0.002 ± 0	0.940 ± 0.06
Rashodia	376.39 ± 24	0.410 ± 0.036	43.436 ± 2.64	55.960 ± 3.79	4.39 ± 0.488	2.62 ± 0.212	1.09 ± 0.088	0.196 ± 0.013	0.006 ± 0	0.75 ± 0.05
Ajwa Al Madinah	290.025 ± 4.6	0.339 ± 0.030	35.941 ± 2.18	53.823 ± 3.65	7.01 ± 0.782	0.37 ± 0.030	0.15 ± 0.013	0.313 ± 0.020	0.001 ± 0	1.200 ± 0.07
Khodry	463.502 ± 6.9	0.564 ± 0.050	59.738 ± 3.63	80.547 ± 5.46	6.52 ± 0.725	0.49 ± 0.040	0.20 ± 0.017	0.291 ± 0.019	0.001 ± 0	1.117 ± 0.07
Khlas Al Ahsa	515.911 ± 7.7	0.637 ± 0.056	67.530 ± 4.11	110.170 ± 7.4	9.06 ± 1.005	0.57 ± 0.046	0.23 ± 0.019	0.404 ± 0.026	0.001 ± 0	1.550 ± 0.10
Sokary	436.75 ± 6.5	0.512 ± 0.045	54.297 ± 3.3	80.640 ± 5.46	6.30 ± 0.701	3.94 ± 0.319	1.64 ± 0.133	0.281 ± 0.018	0.009 ± 0	1.077 ± 0.07
Saffawy	387.4 ± 5.8	0.467 ± 0.041	49.442 ± 3.01	67.377 ± 4.56	5.40 ± 0.601	0.77 ± 0.062	0.32 ± 0.026	0.241 ± 0.015	0.002 ± 0	0.923 ± 0.06
Khlas Al kharj	796.72 ± 31.3	0.919 ± 0.081	97.365 ± 5.92	63.887 ± 4.33	9.37 ± 1.039	0.70 ± 0.057	0.29 ± 0.024	0.418 ± 0.027	0.002 ± 0	1.603 ± 0.10
Mabroom	396.95 ± 15.6	0.479 ± 0.042	50.808 ± 3.09	69.453 ± 4.71	5.85 ± 0.65	0.53 ± 0.043	0.22 ± 0.018	0.261 ± 0.017	0.001 ± 0	1.000 ± 0.06
Khla Al Qassim	665.36 ± 26.1	0.783 ± 0.069	82.930 ± 5.04	57.083 ± 3.87	8.9 ± 0.999	0.65 ± 0.053	0.27 ± 0.022	0.401 ± 0.026	0.002 ± 0	1.537 ± 0.1
Nabtit Ali	180.755 ± 7.1	0.200 ± 0.018	21.141 ± 1.28	30.470 ± 2.06	6.58 ± 0.728	3.95 ± 0.319	1.64 ± 0.133	0293 ± 0.019	0009 ± 0	1.127 ± 0.07
Khals El Shiokh	486.383 ± 19.1	0.581 ± 0.051	61.581 ± 3.74	103.13 ± 6.99	8.6 ± 0.953	0.29 ± 0.024	0.12 ± 0.010	0.383 ± 0.024	0.001 ± 0	1.470 ± 0.09
*p* value	0.00	0.00	0.00	0.00	0.00	0.00	0.00	0.00	0.00	0.00

### 2.2. Principal Component Analysis (PCA) and Hierarchical Clustering (HCA)

**Figure 2:**
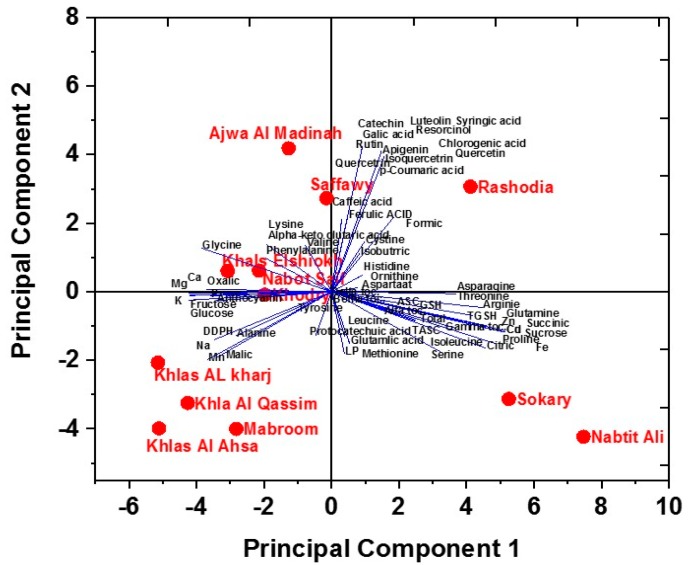
Principal component analysis (PCA) of metabolites in 12 Saudi date cultivars. Bi-plot of principle component 1 (42.7%) and principle component 2 (23.5%).

All the measured metabolites (amino acids, sugars, organic acids, phenolics, flavonoids, antioxidants, and macrominerals and trace elements) were subjected to principal component analysis (PCA) to identify differences in metabolite profiles among the studied date cultivars (Figure 2).

**Figure 3:**
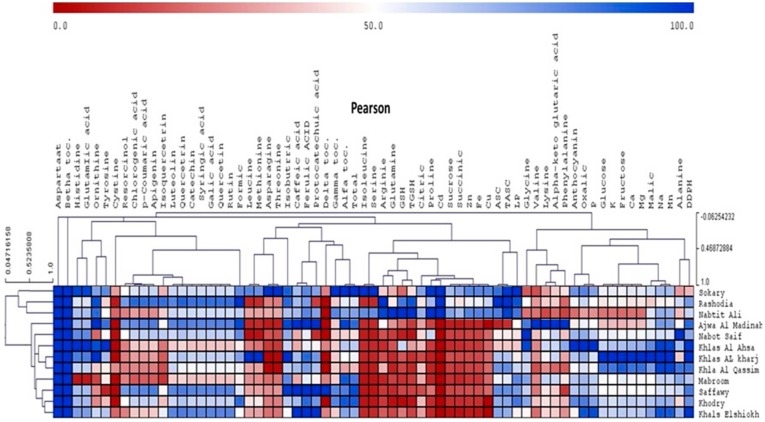
Heat maps of the metabolite profiles of 12 Saudi date cultivars. A total of 42 metabolites were quantified by high performance liquid chromatography for each cultivar, including amino acids, organic acids, sugars, phenolic compounds, glutathione and vitamins.

PCA revealed that the first two principal components (PC1 and PC2) accounted for 49.6% of the total variance within the data set. The first principal component PC1 explained 28.3%, and the second principal component PC2 explained 21.3% of the data variation. These two principal components separated the studied cultivars into four different groups. The first group consisted of Sokary and Nabtit Ali cultivars, the second group of Khlas Al Kharj, Khla Al Qassim, Mabroom, Khlas Al Ahsa, the third group of Khals El Shiokh, Nabot Saif, Khodry, and the fourth group consisted of the Ajwa Al Madinah, Saffawy, Rashodia, Khals El Shiokh, Nabot Saif, and Khodry cultivars. PC1 showed loadings for Sokary and Nabtit Ali, whereas PC2 showed loadings for Ajwa Al Madinah, Saffawy, Khals El Shiokh and Nabot Saif Hierarchical cluster analysis (HCA) of measured metabolites was also performed. A heat map of the metabolite profiling (Figure 3). At a first glance at the entire data set, it is clear that some of the changes in metabolites (aspartate and beta-tocopherol) are almost similar in all cultivars. HCA indicated two main clusters; the first cluster Sokary, Rashodia and Nabtit Ali and the second cluster contained other tested cultivars. This separation could be explained by the lower content of GSH, succinic and citric acids, some amino acids (isoleucine, serine, arginine, proline and glutamine), and some minerals (Cd, Zn, Fe and Cu) in date cultivars of second cluster. Khlas Al Ahsa, Khlas Al kharj, Khlas Al Qassim and Khlas Al Shiokh showed high levels of mono-saccharides (glucose and fructose) and minerals (K, Na, Mg, Mn and Ca), whereas Khlas Al Kharj showed the highest levels. Oppositely, lower levels of resorcinol, chlorogenic acid, coumaric acid, isoquercetin and apignin were recorded in in all Khlas varieties (Al Asha, Al Kharj, El Shiokh).

### 2.3. Biological Activity

#### 2.3.1. DPPH (1,1-Diphenyl-2-picrylhydrazyl) Free Radical Scavenging Activity

DPPH scavenging activity of fruit extracts of different palm date varieties was investigated. Overall, all date varieties showed DPPH scavenging ability (Figure 1a). In particular, Khlas El shiokh, Khlas Al Ahsa, Khlas Al Kharj showed the strongest DPPH scavenging capacity (>38%), whereas Sokary cultivar had the lowest value (14%).

#### 2.3.2. Anti-lipid Peroxidation Assay

**Figure 4:**
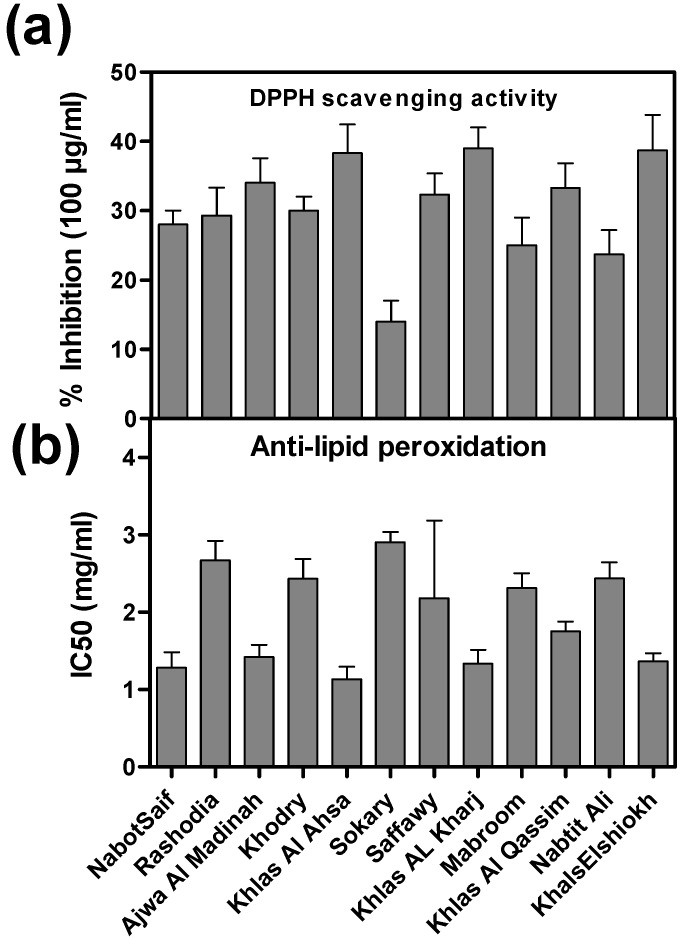
Antioxidant activity: DPPH scavenging activity and anti-lipid peroxidation activity of 12 Saudi date cultivars. (**a**) DPPH (1,1-diphenyl-2-picrylhydrazyl) free radical scavenging activity; (**b**) anti-lipid peroxidation.

Our results showed that almost all the tested palm date extracts inhibited lipid peroxidation (Figure 1b), however extract of Rashodia, Khodry, Sokary, Saffawy, Mabroom and Nabtit Ali showed a higher scavenging potential (*i.e.*, IC_50_ > 2.0). Although Sokary variety showed the highest lipid peroxidation inhibition activity, its DPPH was very low. The higher activity of Sokary extract could be explained by its higher vitamins (ascorbate, tocopherols, Figure 4), total phenolics and flavonoids (Table 4 and Table 5).

### 2.4. Discussion

In this study, the chemical composition and biological activity of 12 date varieties which represent different geographical locations of Saudi Arabia were evaluated. All the measured metabolites (amino acids, sugars, organic acids, phenolics, flavonoids, antioxidants, macrominerals and trace elements) were subjected to principal component analysis (PCA) and hierarchical cluster analysis (HCA) to identify differences in metabolite profiles among cultivars; PCA is widely used to assess the differences between plant varieties/cultivars at the metabolic level. The similarities and variation observed among studied date cultivars in their chemical composition can be explained on the basis of their different metabolic responses and environmental conditions. Indeed metabolic profiles of many crops are significantly affected by genotypes and growing location [30,31,32].

The 2,2-diphenyl-1-picrylhydrazyl (DPPH) radical-scavenging ability assay is widely used to evaluate the free radical scavenging capacity of antioxidants [33]. The decrease in the level of free radicals with the increase in the concentration of the palm date extracts indicates their role extract as antioxidants. High inhibition of DPPH radical formation was also recorded for the Deglet Noor cultivar (about 54%) [34]. The DPPH scavenging ability of date palm extract could be explained by higher availability of different antioxidants. Similar to our results, [7,35] indicated that palm dates contained flavonoids, such as luteolin, quercetin, and apigenin, as well as phenolics, such as *p*-coumaric, ferulic, and sinapic acids, and cinnamic acid derivatives. Accumulation of free radicals can damage cells at the level of nucleic acids, membrane lipids, and proteins, leading to generation of cancer and aging related diseases [36,37]. A strong correlation between the antioxidant activity and the total phenolic and total flavonoids of palm dates was also recorded [38].

The nutritional quality of date palm could, in part, be associated with their major constituents such as flavonoid compounds, phenolics, sugars, amino and organic acids [39,40,41,42,43,44]. Similarly, in our study, higher antimicrobial activity of date palm may be due the presence of phenolics, flavonoids and terpenoids.

Amino acid profiles revealed that all tested date cultivars contained the majority of essential amino acids: lysine, isoleucine, leucine, methionine, threonine, valine, histidine and phenylalanine. This observation was in accordance with those reported by Bouaziz *et al.* [45], where amino acid composition of Tunisian date seeds was profiled; however, lysine presented the largest amount among these Saudi date cultivars. The disappearance of tryptophan could be attributed to its destruction during acid hydrolysis that could also account for the damage to cysteine [46].

Dates contain a high concentration of sugars, which are considered the main component. These carbohydrates are mainly reducing sugars in the form of glucose, fructose, mannose and maltose and non-reducing sugars (primarily sucrose), as well as small amounts of polysaccharides (such as cellulose and starch) [47]. The difference in sugar composition reflects the difference in invertase activity in these cultivars, which causes reduction in sucrose content [3]. Our results are lower than those observed by Al-Farsi *et al.* [16], who studied the compositional and sensory characteristics of three native sun-dried date varieties grown in Oman and reported that total sugar content ranged from 56.1 to 62.2 g/100 g; this is can be explained by non-enzymatic browning during storage (Maillard reaction) [48].

Besides nutritional value, the presence of taste-active components such as organic acids can improve the sensory characteristics of products as they are responsible for the sour, tart, acidic, and characteristic fruity tastes of many foods. Organic acids also influence the growth of microorganisms in fruit and therefore affect the storage quality of the product. Another aspect of organic acids is their influence on the sensory properties of dates. Similarly, the study of Al-Farsi *et al.* [16] indicated that malic acid is the predominant organic acid in dates and has a characteristic fruity, mellow, smooth, tart, and sour taste in fresh fruits. Moreover, the presence and composition of organic acids may be affected by various factors such as variety, growing conditions, maturity, season, geographic origin, fertilization, soil type, storage conditions, amount of sunlight received, and time of harvest, among others [49].

Dates have high content of polyphenolic compounds, which explains their antioxidant activity [7,8]. The amount of total phenolics detected in the tested cultivars came more or less close to that detected by Messaoudi *et al.* [50], who reported that the total phenolic content ranged between 27.2 and 38.5 mg/100 g FW for the methanolic date extracts and between 22.8 and 42.6 mg/100 g FW for the corresponding ethyl acetate extracts, however, Al-Farsi *et al.* [51] reported total phenolics content values between 217.0 to 343.0 mg/100 g fresh weight for some Omani dates. We found nine phenolics *i.e.*, caffeic, ferulic, protocatechuic, catechin, gallic, *p*-coumaric, resorcinol, chlorogenic and syringic acids. Similarly, Al-Farsi *et al.* [51] found nine phenolics in the Omanian date varieties, with *p*-hydroxy-benzoic, protocatechuic, and *m*-coumaric acids as the main components. The heterogeneity of these results could be attributed to several factors—the origin of the plant, the variety, the extraction procedure, and the measurement method [50]. Previous studies also showed that date palm contained flavonoids, such as luteolin, quercetin, and apigenin [38].

Ascorbic acid (ASC), glutathione (GSH) and tocopherol contents in the fruits of the 12 date cultivars were evaluated. Tocopherol also varied significantly among the date cultivars. There are various forms of tocopherols (α, β, γ, δ) and we observed that α-tocopherol was the main contributor for the change in total tocopherol content, as also observed by [52,53]. To our knowledge there are no previous reports measuring ascorbate, glutathione and tocopherol contents in these dates. As we observed significant variation in the contents of antioxidant molecules among the date cultivars, these results have positive implications for breeding programs aimed at increasing the levels of antioxidant compounds in commercial genotypes. These antioxidant molecules play an important role in defense against oxidative stress [54], and also have nutritional value [55].

The results indicated that tested dates cultivars contained significant amounts of minerals, and previous studies [55,56,57] also reported higher contents of minerals, which are three to five times the amounts found in grapes, apples, oranges and bananas [58]. These results confirm that dates have high nutritional value. The high potassium and low sodium contents of dates are suitable for people with hypertension [59]. Similarly, [60,61] reported that dates are a very good source of many minerals which are important for metabolism in human cells. For instance, magnesium and calcium are essential for healthy bone development and for energy metabolism, and iron is essential for red blood cell production.

## 3. Experimental Section

### 3.1. Sample Collection and Extract Preparation

Fresh dates were collected from cultivars grown in the Kingdom of Saudi Arabia. Fruits were collected at the tamr stage from retail local markets in Sakaka city, Aljouf. The local Arabic names of the cultivars used in this study are: Nabot Saif, Rashodia, Ajwa Al Madinah, Khodry, Khlas Al Ahsa, Sokary, Saffawy, Khlas Al Kharj, Mabroom, Khlas Al Qassim, Nabtit Ali and Khals El Shiokh. The dates were washed and stored at −20 °C for further analysis.

### 3.2. Biological Activity

#### 3.2.1. DPPH Free Radical Scavenging Assay

The 1,1-diphenyl-2-picrylhydrazyl (DPPH) free radical scavenging activity of date fruit extracts was estimated according to the method explained by Cheung *et al.* [62] with some modifications. Briefly, the crude extracts were resuspended in methanol, and aliquots of 0.2 mM DPPH in methanol (160 μL) were mixed with each extract (40 μL, 0.01–1 mg·mL^−1^). The mixtures were left under subdued light for 10 min. The absorbance at 520 nm was measured against a blank. The radical scavenging activity was measured as a decrease in the absorbance of DPPH and was calculated using the following equation:

Scavenged DPPH = [1 − (A_sample_ − A_sample blank_)/A_control_ × 100] (1)

where A_control_, A_sample_, and A_sample blank_ represent the absorbance of the control group (160 μL 0.2 mM DPPH and 40 μL methanol or water), sample group (160 μL 0.2 mM DPPH and 40 μL extract or reference compounds (Trolox)), and sample blank (160 μL methanol and 40 μL extract or reference compounds), respectively.

#### 3.2.2. Anti-Lipid Peroxidation Assay

Lipid peroxidation was determined as described by Patro *et al.* [63] with some modifications. Briefly, liposomes were prepared by sonicating a mixture of phosphatidyl choline (300 mg) in 10 mM phosphate buffer (30 mL, pH 7.4) on ice for 2 h. To an aliquot of potassium phosphate buffer (total volume 1 mL) at pH 7.4 (10 mM), the liposomes (250 μL), and extract (resuspended in methanol) or methanol (450 μL), was added FeCl_2_, H_2_O_2_ and ascorbic acid, each to a final concentration of 125 μM. After incubating the mixture at 30 °C for 4 h, 250 μL of the final mixture was added to TCA-TBA-HCl reagent (500 μL, 15% *w*/*v*, TCA; 0.375% *w*/*v*, TBA; 0.25 M HCl). After that the mixture was heated at 100 °C on a boiling water bath for 15 min, followed by centrifugation at 3000 g for 5 min. The absorbance of the supernatant was measured at 532 nm against a blank.

### 3.3. Metabolic Profiling

#### 3.3.1. Amino Acids

Fresh dates were homogenized by using a MagNALyser (Roche, Vilvoorde, Belgium) for 1 min, at 7000 rpm, in 80% (*v*/*v*) aqueous ethanol (1 mL). Samples were spiked with norvaline as a control for the loss of amino acids during extraction. The homogenate was centrifuged at 14,000 rpm for 20 min, the supernatant was evaporated under vacuum, and the pellet was resuspended in chloroform (1 mL). Simultaneously, the residue was reextracted with HPLC grade water (1 mL) using a MagNALyser (Roche, Vilvoorde, Belgium) and the supernatant after centrifugation (14,000 rpm for 20 min) was mixed with the pellet suspended in chloroform. Then they were centrifuged for 10 min at 14,000 rpm and the aqueous phase was filtered using Millipore micro filters (0.2 μM pore size) before assaying free amino acids (FAA) levels. Amino acids were determined by using a Waters Acquity UPLC-tqd system (Milford, Worcester County MA, USA) equipped with a Sinhaa BEH amide 2.1 × 50 column [64].

#### 3.3.2. Sugars

Sugar levels were measured according to Alasalvar *et al.* [65] using high-performance liquid chromatography (HPLC). Sugars were extracted from dates with acetonitrile/water (2 mL, 1:1, *v*/*v*) for 2 min. The extract was then kept in a water bath at 55–60 °C for 15 min (stirring frequently with a glass rod to aid in dissolving the sugars) and subsequently filtered through a Whatman No. 541 filter paper. After that, another 20 mL of solvent was added to the remaining pulp, and the extraction was repeated three times. Finally, all combined supernatants were collected and made up to a final volume of 100 mL with the extraction solvent. Column temperature and injection volume were set at 30 °C and 20 μL, respectively. The mobile phase (filtered through a 0.45 μm Millipore filter and degassed prior to use) was a mixture of acetonitrile and HPLC-grade water at a ratio of 75:25 (*v*/*v*) at 1 mL·min^−1^. Identified sugars were quantified on the basis of peak areas and comparison with a calibration curve obtained with the corresponding standards ranging from 1 to 10 mg/100 mL of acetonitrile/water (1:1, *v*/*v*). Sugars were expressed as milligrams per 100 g of fresh weight (mg/100 g FW).

#### 3.3.3. Organic Acids

Palm fruit samples (500 mg FW) were homogenized in phosphoric acid (0.1%; containing 0.003% butylated hydroxyanisole) by using a MagNALyser. The extract was centrifuged at 14,000 rpm for 30 min at 4 °C. The supernatants were passed through Millipore micro filters (0.2 μM pore size). Organic acids were detected by HPLC using a SUPELCOGEL C-610H column (300 mm × 7.8 mm, Supelco, Sigma, St. Louis, MO, USA) coupled to UV detection system set at 210 nm (LaChrom L-7455 diode array, LaChrom, Tokyo, Japan). The mobile phase was a 0.1% phosphoric acid at a flow rate of 0.45 mL·min^−1^. Organic acids were quantified using a calibration curve obtained with the corresponding standards.

#### 3.3.4. Phenolics

Date fruit samples (50 g DW) were manually separated from the seed, crushed and cut to small pieces with a sharp knife and dry-blended for 3 min with a blender. The date fruit was then extracted with acetone-water (250 mL, 4:1, *v*/*v*), at room temperature for 24 h using an orbital shaker. The extracts were then filtered and centrifuged (Hettich Zentrifugen, Tuttlingen, Germany) at 4000 *g*, for 10 min and the supernatant was concentrated under reduced pressure at 40 °C for 3 h using a rotary evaporator (IKA-WERKE-RV06ML, Stanfer, Germany) to obtain the DPF hydoxyacetone crude extract. The residues were dissolved in HPLC grade MeOH to give 1000 mg·L^−1^ and measured as mentioned previously by Gomaa and AbdElgawad [66]. Briefly methanol-dissolved sample (20 μL) was injected into a Shimadzu HPLC system (SCL-10 A vp, Shimadzu Corporation, Kyoto, Japan). The HPLC system consisted of a diode-array detector and a Lichrosorb Si-60, 7 μm, 3 × 150 mm column. The mobile phase consisted of water/formic acid, 90:10, *v*/*v*; and acetonitrile/water/formic acid, 85:10:5, *v*/*v*/*v*. Tentatively identified phenolic acids and flavonoids were quantified with a calibration curve obtained with the corresponding standards. The results were expressed as mg/100 g DW.

#### 3.3.5. Ascorbate, Glutathione and Tocopherols

Glutathione and ascorbate content was determined by reversed phase HPLC separation, followed by UV detection according to the method described by Potters *et al.* [67]. Total antioxidant concentration (reduced + oxidized) was determined after reduction with 0.04 M DTT for 10 min at room temperature, and the redox status was calculated as the ratio of the reduced form to the total concentration. Tocopherols were extracted with hexane, and measured according to the method of Siebert 1999 [68]. The extract was vaccum dried (CentriVap concentrator, Labconco, KS, USA) and was re-suspended in hexane. Tocopherols were separated and quantified by HPLC (Shimadzu, Hertogenbosch, Netherlands) using normal phase conditions (Particil Pac 5 μm column material, length 250 mm, i.d. 4.6 mm). 5,7-dimethyltocol (DMT; 5 ppm) was used as an internal standard. Data were analyzed with Shimadzu Class VP 6.14 software provided by the HPLC system (Shimadzu, Tokyo, Japan).

#### 3.3.6. Macro-Minerals and Trace Elements

Date fruits were digested in a 5:1 ratio of HNO_3_/H_2_O in an oven and macro-minerals and trace elements were determined by mass spectrometry (ICP-MS, Finnigan Element XR, Scientific, Bremen, Germany) according to Agusa *et al.* [69]. A mixture of standards was prepared in 1% nitric acid.

### 3.4. Statistical Analysis

The data were analyzed by procedure of the Statistical Analysis System (SPSS Inc., Chicago, IL, USA). The assumptions of normality of distribution and homogeneity of variance were examined. Since both assumptions were met, transformations were not necessary and analysis of variance (ANOVA) was done on the original data. The differences between cultivars were tested by one-way ANOVA procedure. Number of replicates for each cultivar were three (*n* = 3). The significant differences between the means were determined by using the Duncan test (*p* < 0.05). Principal Component Analysis (PCA) was performed by using OriginLab software (9, OriginLab, Northampton, MA, USA). Cluster analysis was performed by using Pearson distance metric by using MultiExperiment Viewer (MeV)™ 4 software package (version 4.5, Dana-Farber Cancer Institute, Boston, MA, USA). All parameters and cultivars were included in the analysis.

## 4. Conclusions

The differences in the chemical composition of date fruits altered their nutritional value and biological activities. Our study showed that the date fruit extracts from different cultivars have different free radical scavenging and anti-lipid peroxidation activities. The similarities and chemical composition variations observed among the studied date cultivars could explained the variation in date fruit biological activity. Future studies exploring the link between dates’ nutritional quality and their growth climate conditions are needed.
